# Determinants of acquired disability and recovery from disability in Indian older adults: longitudinal influence of socio-economic and health-related factors

**DOI:** 10.1186/s12877-021-02372-x

**Published:** 2021-07-16

**Authors:** Ronak Paul, Shobhit Srivastava, T. Muhammad, Rashmi Rashmi

**Affiliations:** International Institute for Population Sciences, Maharashtra 400088 Mumbai, India

**Keywords:** Activities of daily living, Change in disability status, Recovery from disability, Incidence of disability, Elderly, India Human Development Survey

## Abstract

**Background:**

There is a higher burden of functional disability for Indian older adults with substantial variations across different geographic regions and socioeconomic groups as compared to other ageing Asian countries. Thus, using a national sample of older adults aged 60+ years, we aim to explore how common is acquiring of disability and recovery from disability among the older population of a developing country like India, and how do the various socioeconomic and health-related conditions impact this transition in disability status.

**Method:**

The current study uses two waves of the India Human Development Survey (IHDS) and is based on panel data of 10,527 older adults. Both bivariate and multiple variable regression analysis were performed using two binary outcome variables in this study – whether older adults acquired disability and recovered from disability between round-I and round-II, respectively.

**Results:**

Nearly 31.5% and 4.4 % of older adults have acquired and recovered disability across the two rounds respectively. About 38.5%  and 45.8 % of female older adults’ disability status changes to disable and recovered in round-II respectively. A lesser proportion of older adults have recovered from a disability who have a chronic disease in round-I. Cataract chronic conditions among older adults in round-I had shown 1.45 times (CI: 1.07–1.97) a significantly higher likelihood of acquiring disability in round-I. Older adults who were unmarried and were not working in round-I have 1.12 times (CI: 1.01–1.25) and 1.21 times (CI: 1.06–1.39) higher likelihood of acquiring disability in round-II respectively. Recovering from disability was mostly seen among older adults who belong to the richest (OR: 2.38, CI: 1.31–4.33) and medium (OR: 2.16, CI: 1.27–3.69) wealth quintile households. Older adults residing in the central region of India have 2.72 times (CI: 2.31–3.19) significantly higher chance of acquiring disability than those who were residing in northern regions.

**Conclusion:**

Appropriate measures are required to highlight the importance of chronic physical diseases and several socio-demographic factors that may negatively affect the trajectory of disability in older ages.

## Background

Due to the on-going demographic transition towards an ageing population, there is an increasing trend in the prevalence of late-life disabilities especially in developing countries [1, 2]. Disability in old age is common and becomes a stress factor for individual’s family as well as the society at large due to the limited resources for assistance, care and rehabilitation [3, 4]. While experiencing a double burden of both communicable and non-communicable diseases [5], three-fourth of the Indian population aged 50 and above are suffering from some form of disability [6]. At the same time, a positive relationship between disability and poverty in low and middle income countries has been documented in the recent literature [7].

Multiple studies found that older age, female sex, being widowed or single, sedentary physical activity, obesity, smoking, or having chronic diseases are commonly associated with disability [8–10]. The literature on gender differences in functional limitations and disabilities has shown a female disadvantage [11–13]. Besides, older people with low socioeconomic status, lower levels of education, less engaged in social activities, not in the working force, and have poor health status were more likely to have disabilities [14–17]. Studies based on World Health Organization (WHO) Study on Global Ageing and adult health (SAGE) data have explored the association between socio-demographic factors, chronic diseases, and disability [13, 18–20], and found education and employment-driven inequalities in the prevalence of disability and its concentration in the rural areas of the developing countries. However, a review of resilience literature talking about the ability of older individuals to maintain a positive mental state during exposure to health problems shows that those living in rural areas might show signs of high resilience by overcoming socioeconomic obstacles and being able to live a decent life despite declining health and the presence of multiple functional limitations [21].

Furthermore, factors associated with no recovery from disability were chronic diseases, depression, older age, female gender, and low educational level [22]. Similarly, in a community-based study, a greater number of limitations in functional health was shown as a predictor of failing to recover from disabilities in old age [23]. On the other hand, a recent study among older Mexican adults suggested that older individuals with several chronic conditions such as heart disease at baseline were also associated with an improvement or a recovery from disabilities [24]. Findings also suggest that older adults with illnesses are often forced to alter their lifestyle and prefer better nutrition, increase physical activity and social participation and reduce or quit alcohol consumption and tobacco use thus improving their well-being and resulting in recovery from disabilities [25, 26].

It is however demonstrated that older women have a lower probability than older men of recovering after reporting functional limitations [27]. The study also revealed that living alone and other socio-demographic factors that include caste and family structure played a significant role in disability dynamics [28, 29]. Further, several studies in India have demonstrated a higher likelihood of being sick and disabled among older adults who are economically disadvantaged and belonged to lower caste groups [30, 31]. It is again observed that currently married status and living with spouse and children were negatively associated with the prevalence of disability or remaining disabled [32–35].

Besides, a higher burden of functional disability for Indian older adults with substantial variations across different geographic regions and socioeconomic groups has been reported in multiple studies as compared to other ageing Asian countries [29, 36–38]. Although a couple of studies observed the greater socioeconomic inequalities in disability among older Indian adults [11, 39] and their treatment seeking behaviour [40], a better knowledge of the determinants of changes in the disability status will be helpful from the perspective of targeting the most vulnerable groups and establishing preventive health priorities among the older population. Since existing studies on physical limitations highlight the determinants of late-life disabilities, we focus on the associated factors of recovery from or acquiring of disability in old age. Thus, using a national sample of older Indian adults aged 60+, the present study aims to explore how common is acquiring of disability and recovery from disability among the older population of a developing country like India, and how do the various socioeconomic and health-related conditions impact this transition in disability status.

## Data, Variables and Methods

### Data source

This study used round-I and round-II of the India Human Development Survey (IHDS). IHDS round-I, conducted during 2005, is a nationally representative and multi-topic survey of 41,554 households across all the states and union territories of India except for Andaman & Nicobar Islands and Lakshadweep [41]. IHDS round-II, conducted in 2012, is also a nationally representative and multi-topic survey of 42,152 households with geographical coverage similar to round-I [42]. IHDS round-II re-interviewed 83 % of the households from round-I. Both rounds of IHDS adopted a multistage stratified random sampling design. Further details regarding the IHDS sampling frame, data collection procedure and respondent consent can be found elsewhere [43, 44].

This study is based on older adults aged 60 + years who participated in both rounds of IHDS. During IHDS round-I (baseline survey), there were 17,904 individuals and among them, 4736 older adults were not alive and 2641 older adults were lost to follow-up during round-II. Therefore, our current study is based on panel data of 10,527 older adults. Additionally, there were no records with missing information for all the variables used in our study.

### Outcome variables

We used two binary outcome variables in this study – whether older adults acquired disability and recovered from disability between round-I and round-II, respectively. Both these outcome indicators were obtained from an older adult’s self-reported disability status during both rounds of IHDS. During both rounds, IHDS asked respondents that whether they had difficulty in – “walking 1 km”, “going to the toilet without help”, “dressing without help”, “hearing normal conversation”, “speaking normally”, “seeing distant things” and “seeing near objects such as reading/sewing”. Responses to these seven questions were coded as “0” (No difficulty), “1” (With difficulty) and “2” (Unable to do it). We summed the coded responses for each person to obtain a disability score ranging from 0 to 14 and found the median disability score to be 0 in both rounds. Therefore, older adults with a score of 0 were classified as “not disabled” and with a score above 0 were classified as “disabled”.

In the acquired disability variable (no, yes), among the older adults who were not disabled in 2005, those were disabled during 2012 were categorized as “yes” and those who were not disabled during both 2005 and 2012 were categorized into “no” respectively. Equivalently, in the recovered from disability variable (no, yes), older adults who were disabled in 2005 but were not disabled during 2012 were categorized as “yes” and those who were disabled in both rounds were categorized into “no”.

### Independent variables

Existing studies have shown several factors, which influences the change in disability status among older adults. We controlled for the effects of the majority of these explanatory variables, conditional to their availability in the IHDS dataset. The demographic and social control variables related to the older adults include – gender of individual (male, female), age group (60–69 years, 70–79 years, 80 + years), current marital status (currently married, currently not married), level of education (no formal schooling, less than 5 years of schooling, 6–10 years of schooling, more than 10 years of schooling), current work status (currently working, currently not working). Additionally, we included self-reported indicators of chronic illness indicating whether an older adult has – cardiovascular diseases (no, yes), hypertension (no, yes), diabetes (no, yes), respiratory illness (no, yes), cataract (no, yes) and any other chronic illness other than the above (no, yes). We also controlled for relevant household socio-economic characteristics – headship status (no, yes), family structure (joint/extended, nuclear, single generation), wealth quintile (poorest, poor, medium, rich, richest), household below poverty line (BPL) status (no, yes), the caste of household (scheduled tribes (ST), scheduled castes (SC), other backward classes (OBC), others), the religion of household (Hindu, Muslim, others), place of residence (urban, rural). Additionally, we also included the country region that a person comes from (northern, north-eastern, central, eastern, western, southern). All the above characteristics were measured for the older adults during round-I.

During round-I IHDS collected information on the marital status of each person and originally categorized them into six categories – “spouse absent”, “married”, “single”, “widowed”, and “separated/divorced”. Owing to the skewed population distribution across each category, we have recoded the original variable into a binary marital status variable (currently married, currently not married). Here, all individuals not included in the “married” category in the original variable were included in the “currently not married” category of the recoded variable.

During round-I, IHDS obtained the information on whether each person reportedly suffered from – cataract, tuberculosis, hypertension, cardiovascular diseases, diabetes, leprosy, cancer, asthma, polio, paralysis, epilepsy, mental illness, sexually transmitted diseases (STD) and any other chronic disease. If an older adult suffered from the above chronic diseases, then they were coded as “yes” and otherwise they were coded as “no”.

Household family structure was obtained from the information given on the relationship of each household member with the head of the household. Based on this information we classified the family structure into – single generation, nuclear and joint/extended family. The single generation includes a married/cohabiting couple or a single person household. The nuclear family includes married/cohabiting partners along with their dependent and unmarried children. The joint family includes a parent and/or partner along with their children and grandchildren. The extended family is similar to a joint family structure with the exception that it also includes “extended members”, that is, people who are not directly related to the household head by blood.

The household wealth quintile for round-I was calculated using principal component analysis [45]. We generated a wealth score for each household using the available information on household asset ownership, livestock ownership, building material used in household, household water source, household sanitation facility and the number of rooms. Based on the wealth score we categorized the households into five categories (poorest, poor, medium, rich, and richest).

IHDS classified caste of the household head into five categories (Brahmin, Other Backward Caste (OBC), Schedule Caste (SC), Schedule Tribe (ST), Others). We recoded the caste variable into four categories – “OBC”, “SC”, “ST” and “Other”, where the “Other” category consists of “Brahmin” and “Other” categories from the original variable. The people in the ST and SC category belong to the most socially backward group of people who historically belonged to the lower rung of the now constitutionally-abolished Indian caste system. People in the OBC category, as the name suggests, also belong to a group of the socially and economically backward population, with conditions better than that of the SC/ST population. The “Other” category consists of all people who do not belong to either of the three caste groups.

The country regions during round-I were formed by grouping the erstwhile 33 states and union territories of India into six regions. The northern region includes Chandigarh, Delhi, Haryana, Himachal Pradesh, erstwhile Jammu & Kashmir, Punjab, Uttaranchal and Rajasthan. The north-eastern region includes Assam, Arunachal Pradesh, Manipur, Meghalaya, Mizoram, Nagaland, Tripura and Sikkim. The central region consists of Madhya Pradesh and Chhattisgarh. The eastern region includes Bihar, Jharkhand, Odisha and West Bengal. The western region comprises Dadra & Nagar Haveli, Daman & Diu, Goa, Gujarat and Maharashtra. The southern region comprises erstwhile Andhra Pradesh, Karnataka, Kerala, Tamil Nadu and Pondicherry.

### Statistical methods

We performed bivariate and multivariate analysis to achieve the study objectives. Bivariate and multivariate analysis was performed in two sets by taking “acquired disability” and “recovered from disability” as the outcome variable respectively. The first set involved those older adults who were not disabled in round-I, and among them, we examined the factors responsible for acquiring disability in round-II. The second set examined the factors associated with recovery from disability in round-II among those older adults who were disabled in round-I. Owing to the binary nature of the outcome variable, bivariate analysis was done using the chi-square test for association. Equivalently, multivariate analysis was performed by estimating multivariable logistic regression models. Odds ratios in the multivariable models show the association between the outcome variables of change in disability status and the predictor variables. The odds ratio for the first set, measures the odds of acquiring disability relative to having no disability among the older adults belonging to a particular category of an explanatory variable given the effect of all the other explanatory variables remain constant [46]. Similarly, the odds ratio for the second set, gives the odds of recovery from disability relative to the older adults who had disability given the effect of the other independent variables are constant. The odds ratio can take any value above zero, with a value between 0 and 1 denoting a negative association, and a value more than 1 denoting a positive association.

Additionally, we checked for multicollinearity in both the regression models and found the mean value of the variance inflation factor (VIF) to be less than 1.35. Thus, our estimated regression models do not suffer from multicollinearity. All the statistical estimations were performed using the STATA software version 14.2 [47].

## Results

### Descriptive Statistics

**Fig. 1:**
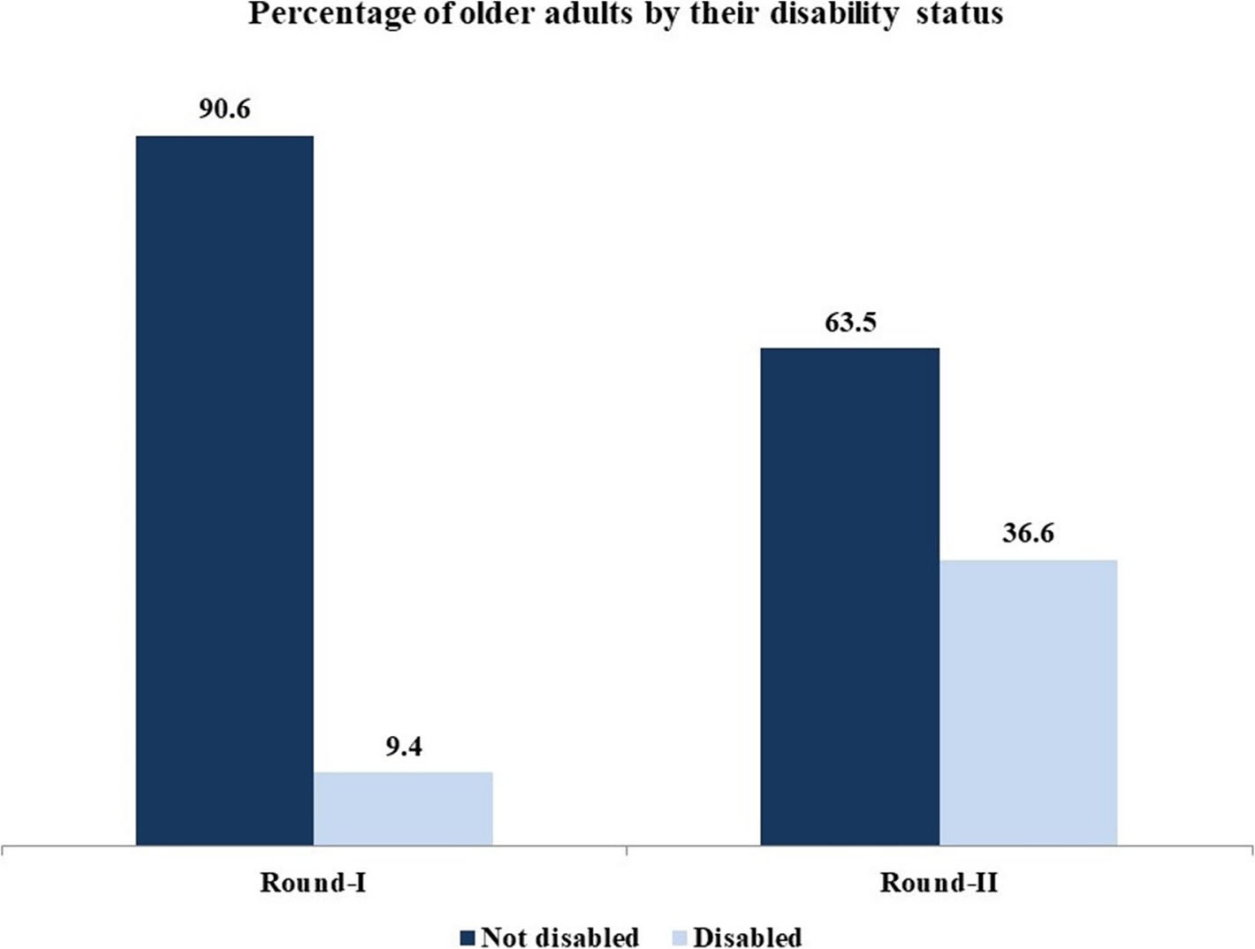
Percentage distribution of older adults (60 + years) by the change in disability status from round-I to round-II

**Fig. 2:**
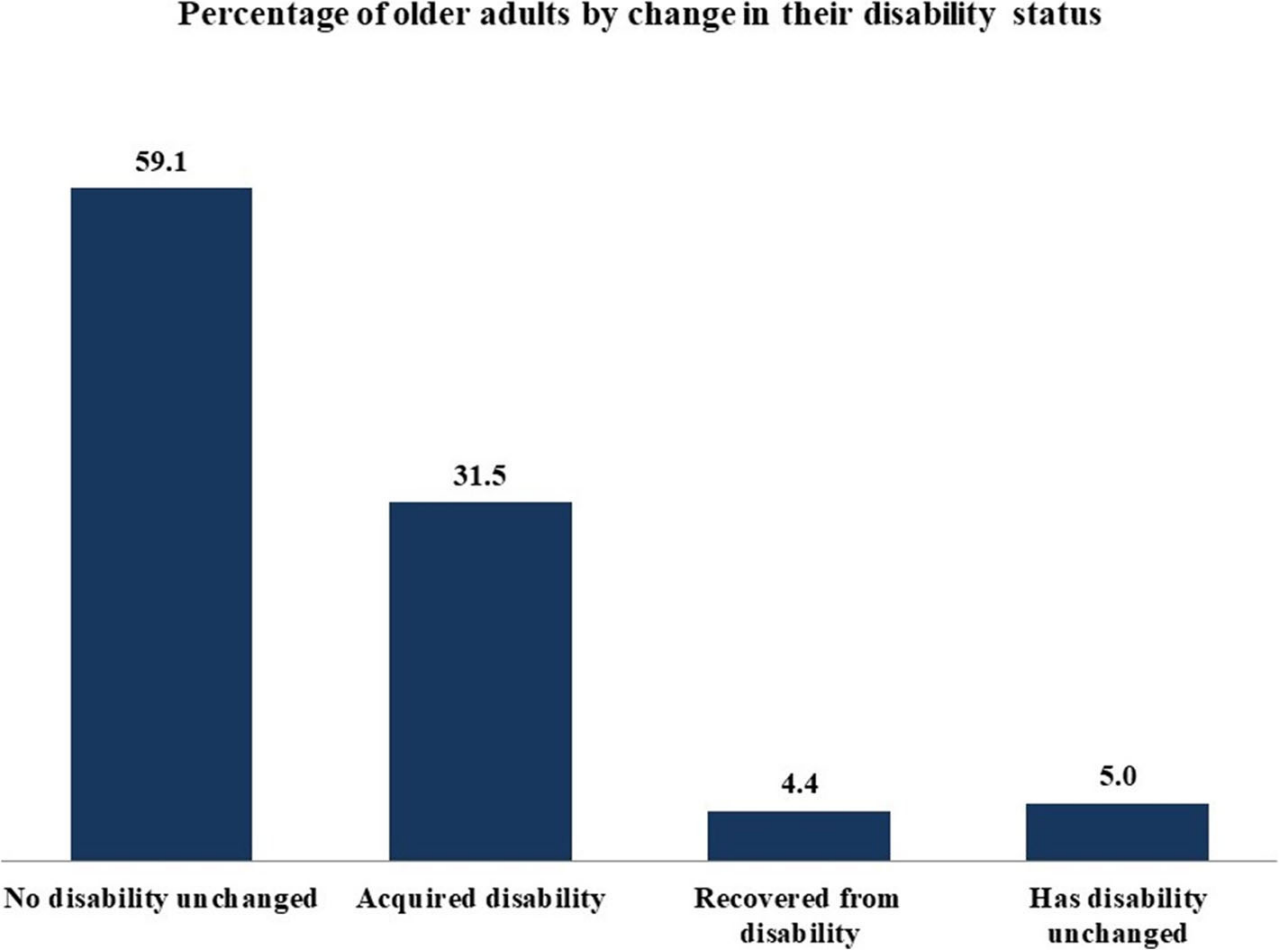
Percentage distribution of older adults (60 + years) by the disability status during round-I and round-II

Figure 1 provides a cross-sectional view of disability status among older adults in India. In the IHDS panel, we see that 9% of older adults had a disability in 2005, however, the percentage increased to 37% in 2012. Figure 2 presents the change in disability status of older adults across the two rounds of IHDS. Results show that 32% and 4 % of older adults had acquired disability and recovered from disability across rounds-I and -II respectively.

Table 1 shows the characteristics of 10,527 older adults in the IHDS panel dataset during 2005. Among the panel of older adults, we found that 52 % were females and 33 % were not married during round-I. Further, six in every ten older adults had no formal schooling and 15 % were working during 2005. Moreover, half of the older adults were household heads and three-fourth lived in a joint/extended family. Nearly 32% and 20 % of older adults lived in the lowest 40 % wealth quintile and below poverty line households respectively. Furthermore, one in every five older adults belonged to the SC/ST caste and 74 % lived in rural communities. Additionally, 33 % of older adults resided in the northern Indian states followed by 24 % of older adults in the southern region.

**Table 1:** Absolute (N) and percentage (%) distribution of older adults (60 + years) by relevant socio-economic and demographic characteristics across the full and panel sample during round-I (baseline period) in India

Characteristics	IHDS round-I (Baseline Period)				Absolute difference
	**Full Sample**		**Panel Sample**		
	**N**	**%**	**N**	**%**	**%**
**Gender of individual**					
Male	8,963	50.1	5,076	48.2	1.9
Female	8,941	49.9	5,451	51.8	1.9
**Age-group (in years)**					
60–69	10,917	61.0	7,343	69.8	8.8
70–79	5,200	29.0	2,663	25.3	3.7
80+	1,787	10.0	521	4.9	5.1
**Current marital status**					
Currently married	11,254	62.9	7,086	67.3	4.4
Currently not married	6,650	37.1	3,441	32.7	4.4
**Level of education**					
No formal schooling	10,672	59.6	6,262	59.5	0.1
Less than 5 years of schooling	3,128	17.5	1,867	17.7	0.2
6–10 years of schooling	2,736	15.3	1,622	15.4	0.1
More than 10 years of schooling	1,368	7.6	776	7.4	0.2
**Current work status**					
Currently working	2,407	13.4	1,593	15.1	1.7
Currently not working	15,497	86.6	8,934	84.9	1.7
**Household headship status**					
No	9,294	51.9	5,334	50.7	1.2
Yes	8,610	48.1	5,193	49.3	1.2
**Family structure**					
Joint/Extended	13,843	77.3	8,109	77.0	0.3
Nuclear	2,016	11.3	1,268	12.0	0.7
Single generation	2,045	11.4	1,150	10.9	0.5
**Household wealth quintile**					
Poorest	2,699	15.1	1,603	15.2	0.1
Poor	2,961	16.5	1,777	16.9	0.4
Medium	3,491	19.5	2,109	20.0	0.5
Rich	3,852	21.5	2,241	21.3	0.2
Richest	4,901	27.4	2,797	26.6	0.8
**Household below poverty line (BPL)**					
No	14,312	79.9	8,445	80.2	0.3
Yes	3,592	20.1	2,082	19.8	0.3
**Caste of household**					
Scheduled Tribes	1,126	6.3	607	5.8	0.5
Scheduled Castes	3,167	17.7	1,858	17.6	0.1
Other Backward Classes	7,191	40.2	4,334	41.2	1.0
Others	6,420	35.9	3,728	35.4	0.5
**Religion of household**					
Hindu	14,641	81.8	8,676	82.4	0.6
Muslim	1,758	9.8	994	9.4	0.4
Others	1,505	8.4	857	8.1	0.3
**Participates in social groups**					
Yes	6,362	35.5	3,813	36.2	0.7
No	11,542	64.5	6,714	63.8	0.7
**Place of residence**					
Urban	5,257	29.4	2,764	26.3	3.1
Rural	12,647	70.6	7,763	73.7	3.1
**Country regions**					
Northern	5,771	32.2	3,488	33.1	0.9
North Eastern	562	3.1	290	2.8	0.3
Central	1,728	9.7	1,029	9.8	0.1
Eastern	2,637	14.7	1,599	15.2	0.5
Western	2,577	14.4	1,584	15.0	0.6
Southern	4,629	25.9	2,537	24.1	1.8
**Overall**	**17,904**	**100**	**10,527**	**100**	**0**

***Note – (a) Full sample refers to the original population surveyed during the baseline period (round-I); (b) Panel sample refers to the population who were alive in both rounds of IHDS***

We compared the percentage distribution of the population of older adults across selected characteristics for both the full sample and panel sample in round-I (baseline), as shown in the last column of Table 1. Indeed, the percentage distribution across the individual-related, household-related and socioeconomic characteristics were similar among both datasets. Only the percentage distribution by age group differed by more than 5 % between the two datasets.

### Bivariate Analysis

The first part of Table 2 shows the association of different bio-demographic and socio-economic characteristics of older adults in round-I with their acquired disability status during round-II. Among 9533 older adults who were not disabled in round-I, 35 % acquired disability in round-II. We observed that female older adults (38 %) and elderly in the age group of 80 + years (52 %) were highly likely to acquire disability during round-II. Further, older adults who had hypertension, cataract, or any other chronic condition had greater likelihood of acquired the disability. Moreover, 40 % of unmarried older adults and 37 % older adults who were not head of the household became disabled between rounds-I and -II. Furthermore, acquiring disability was more common among older adults residing in a single generation family (42 %), poorest wealth quintile (39 %) and below poverty line households (37 %). The proportion of older adults becoming disabled in round-II was highest in the Central region (58 %) followed by the Southern (41 %) and Northern (34 %) regions respectively.

**Table 2:** Bivariate analysis showing the association of bio-demographic and socio-economic characteristics in round-I with the acquiring and recovery from disability in round-II respectively among panel data of older adults (60 + years) in India

Characteristics	Change in disability status during round-II								
	**Total**	**Disability acquired**				**Total**	**Disability recovered**		
	**N**	**N**	**%**	✗2 tests		**N**	**N**	**%**	✗2 tests
**Gender of individual**									
Male	4,654	1,440	30.9	✗2 = 59.56; *p*-value = 0.001		422	201	47.6	✗2 = 0.32; *p*-value = 0.568
Female	4,879	1,877	38.5			572	262	45.8	
**Age-group (in years)**									
60–69	6,755	2,102	31.1	✗2 = 152.14; *p*-value = 0.001		588	303	51.5	✗2 = 14.85; *p*-value = 0.001
70–79	2,331	985	42.3			332	134	40.4	
80+	447	230	51.5			74	26	35.1	
**Had Cardiovascular diseases**									
No	9,400	3,263	34.7	✗2 = 2.00; *p*-value = 0.157		952	448	47.1	✗2 = 2.08; *p*-value = 0.149
Yes	133	54	40.6			42	15	35.7	
**Had Hypertension**									
No	9,030	3,115	34.5	✗2 = 6.73; *p*-value = 0.009		825	389	47.2	✗2 = 0.64; *p*-value = 0.424
Yes	503	202	40.2			169	74	43.8	
**Had Diabetes**									
No	9,276	3,217	34.7	✗2 = 1.97; *p*-value = 0.160		896	424	47.3	✗2 = 2.01; *p*-value = 0.156
Yes	257	100	38.9			98	39	39.8	
**Had Respiratory illnesses**									
No	9,290	3,227	34.7	✗2 = 0.55; *p*-value = 0.457		935	438	46.8	✗2 = 0.45; *p*-value = 0.504
Yes	243	90	37.0			59	25	42.4	
**Had Cataract**									
No	9,346	3,231	34.6	✗2 = 10.53; *p*-value = 0.001		867	408	47.1	✗2 = 0.63; *p*-value = 0.429
Yes	187	86	46.0			127	55	43.3	
**Had Any other chronic illnesses**									
No	9,160	3,159	34.5	✗2 = 9.79; *p*-value = 0.002		859	412	48.0	✗2 = 4.86; *p*-value = 0.027
Yes	373	158	42.4			135	51	37.8	
**Current marital status**									
Currently married	6,478	2,097	32.4	✗2 = 52.34; *p*-value = 0.001		608	287	47.2	✗2 = 0.24; *p*-value = 0.620
Currently not married	3,055	1,220	39.9			386	176	45.6	
**Level of education**									
No formal schooling	5,724	2,091	36.5	✗2 = 32.35; *p*-value = 0.001		538	263	48.9	✗2 = 4.04; *p*-value = 0.257
Less than 5 years of schooling	1,646	575	34.9			221	93	42.1	
6–10 years of schooling	1,452	417	28.7			170	74	43.5	
More than 10 years of schooling	711	234	32.9			65	33	50.8	
**Working status**									
Working	1,505	466	31.0	✗2 = 11.56; *p*-value = 0.001		88	38	43.2	✗2 = 0.45; *p*-value = 0.503
Not working	8,028	2,851	35.5			906	425	46.9	
**Household headship status**									
No	4,803	1,792	37.3	✗2 = 26.99; *p*-value = 0.001		531	242	45.6	✗2 = 0.46; *p*-value = 0.496
Yes	4,730	1,525	32.2			463	221	47.7	
**Family structure**									
Joint/Extended	7,369	2,576	35.0	✗2 = 43.23; p-value = 0.001		740	342	46.2	✗2 = 1.71; p-value = 0.425
Nuclear	1,169	328	28.1			99	52	52.5	
Single generation	995	413	41.5			155	69	44.5	
**Household wealth quintile**									
Poorest	1,468	570	38.8	✗2 = 17.31; *p*-value = 0.002		135	54	40.0	✗2 = 4.74; *p*-value = 0.315
Poor	1,633	591	36.2			144	66	45.8	
Medium	1,913	633	33.1			196	102	52.0	
Rich	2,023	670	33.1			218	101	46.3	
Richest	2,496	853	34.2			301	140	46.5	
**Household below poverty line (BPL)**									
No	7,619	2,605	34.2	✗2 = 6.10; *p*-value = 0.013		826	385	46.6	✗2 = 0.01; *p*-value = 0.966
Yes	1,914	712	37.2			168	78	46.4	
**Caste of household**									
Scheduled Tribes	563	177	31.4	✗2 = 16.89; *p*-value = 0.001		44	26	59.1	✗2 = 5.92; *p*-value = 0.116
Scheduled Castes	1,723	613	35.6			135	71	52.6	
Other Backward Classes	3,936	1,447	36.8			398	175	44.0	
Others	3,311	1,080	32.6			417	191	45.8	
**Religion of household**									
Hindu	7,897	2,787	35.3	✗2 = 5.39; *p*-value = 0.068		779	367	47.1	✗2 = 5.42; *p*-value = 0.067
Muslim	898	285	31.7			96	51	53.1	
Others	738	245	33.2			119	45	37.8	
**Participates in social groups**									
Yes	3,415	1,138	33.3	✗2 = 5.08; *p*-value = 0.024		398	165	41.5	✗2 = 6.99; *p*-value = 0.008
No	6,118	2,179	35.6			596	298	50.0	
**Place of residence**									
Urban	2,512	838	33.4	✗2 = 3.09; *p*-value = 0.078		252	133	52.8	✗2 = 5.21; *p*-value = 0.022
Rural	7,021	2,479	35.3			742	330	44.5	
**Country regions**									
Northern	3,245	1,090	33.6	✗2 = 433.49; *p*-value = 0.001		243	121	49.8	✗2 = 48.32; *p*-value = 0.001
North Eastern	284	22	7.7			6	4	66.7	
Central	937	539	57.5			92	28	30.4	
Eastern	1,495	409	27.4			104	69	66.3	
Western	1,412	365	25.8			172	99	57.6	
Southern	2,160	892	41.3			377	142	37.7	
**Overall**	**9,533**	**3,317**	**34.8**			**994**	**463**	**46.6**	

Note – (a) ✗ ^*2*^-tests denotes the Chi-square test for association

Similarly, the second part of Table 2 shows the association of explanatory characteristics in round-I with the recovery of disability among older adults during round-II. Among 994 older adults who were disabled in round-I, 47 % recovered from their disability between round-I and round-II. Recovery from disability was higher among older adults (52 %) in the 60–69 age group. Although the proportion of recovery from disability was higher among older adults who had no chronic diseases during round-I, the bivariate association was not statistically significant at the 5 % level. Moreover, 42 % of older adults who did not participate in social groups and 53 % elderly living in urban communities recovered from disability between rounds-I and -II. Additionally, the proportion of older adults recovering from disability was highest in the North-eastern region (67 %) followed by the Eastern (66 %) and Western (58 %) regions respectively.

### Multivariate Analysis

Table 3 show the results from logistic regression models indicating the association of explanatory characteristics of in round-I with the transition of disability status in older adults between rounds-I and -II. We see that, female older adults had 1.40 times (CI: 1.21–1.61) significantly higher odds of acquiring disability in round-II. Moreover, with the growing age the likelihood of acquiring disability among older adults increases; and their chance of recovering from disability decreases significantly. Further, we observed that older adults with cataract chronic conditions in round-I had 1.45 times (CI: 1.07–1.97) higher odds of acquiring disability by round-II. Older adults who were not married and were residing in single generation family in round-I have 1.12 times (CI: 1.01–1.25) and 1.35 times (CI: 1.16–1.57) greater likelihood of acquiring disability by round-II respectively. Notably, recovering from disability was mostly seen among older adults who belonged to the richest (OR: 2.38, CI: 1.31–4.33), rich (OR: 2.03, CI: 1.16–3.53) and medium (OR: 2.16, CI: 1.27–3.69) wealth quintile households in comparison to their counterparts in the poorest wealth quintile. Older adults residing in the Central region of India have 2.72 times (CI: 2.31–3.19) significantly higher chance of acquiring disability than those who were residing in Northern regions. Additionally, recovery from disability was higher among older adults in the Eastern (OR: 2.70, CI: 1.59–4.56) and Western (OR: 1.83, CI: 1.18–2.82) regions of India respectively compared to the residents of the Northern region.

**Table 3:** Odds ratio from logistic regression models showing the association of bio-demographic and socio-economic characteristics measure in round-I with acquiring and recovery from disability during round-II among panel data of older adults

Characteristics	Change in disability status during round-II			
	**Disability acquired**		**Disability recovered**	
	**OR**	**95 % CI**	**OR**	**95 % CI**
**Gender of individual**				
Male	1.00		1.00	
Female	1.40***	(1.21–1.61)	0.85	(0.56–1.29)
**Age-group (in years)**				
60–69	1.00		1.00	
70–79	1.60***	(1.45–1.78)	0.57***	(0.42–0.77)
80+	2.27***	(1.85–2.78)	0.41***	(0.23–0.72)
**Had Cardiovascular diseases**				
No	1.00		1.00	
Yes	1.32	(0.91–1.91)	0.59	(0.30–1.19)
**Had Hypertension**				
No	1.00		1.00	
Yes	1.16	(0.95–1.42)	1.10	(0.74–1.62)
**Had Diabetes**				
No	1.00		1.00	
Yes	1.14	(0.86–1.50)	0.74	(0.45–1.21)
**Had Respiratory illnesses**				
No	1.00		1.00	
Yes	1.04	(0.79–1.37)	0.93	(0.53–1.66)
**Had Cataract**				
No	1.00		1.00	
Yes	1.45**	(1.07–1.97)	1.15	(0.76–1.76)
**Had Any other chronic illnesses**				
No	1.00		1.00	
Yes	1.36***	(1.09–1.69)	0.57***	(0.38–0.85)
**Current marital status**				
Currently married	1.00		1.00	
Currently not married	1.12**	(1.01–1.25)	1.06	(0.76–1.47)
**Level of education**				
No formal schooling	1.00		1.00	
Less than 5 years of schooling	1.06	(0.93–1.21)	0.76	(0.52–1.10)
6–10 years of schooling	0.94	(0.81–1.10)	0.72	(0.45–1.13)
More than 10 years of schooling	1.01	(0.83–1.22)	0.82	(0.45–1.52)
**Working status**				
Working	1.00		1.00	
Not working	1.21***	(1.06–1.39)	1.34	(0.80–2.23)
**Household headship status**				
No	1.00		1.00	
Yes	1.09	(0.95–1.25)	0.94	(0.63–1.38)
**Family structure**				
Joint/Extended	1.00		1.00	
Nuclear	0.93	(0.80–1.09)	1.17	(0.72–1.88)
Single generation	1.35***	(1.16–1.57)	1.07	(0.71–1.62)
**Household wealth quintile**				
Poorest	1.00		1.00	
Poor	0.93	(0.80–1.09)	1.58*	(0.92–2.72)
Medium	0.83**	(0.71–0.97)	2.16***	(1.27–3.69)
Rich	0.88	(0.75–1.04)	2.03**	(1.16–3.53)
Richest	0.92	(0.76–1.11)	2.38***	(1.31–4.33)
**Household below poverty line (BPL)**				
No	1.00		1.00	
Yes	1.02	(0.91–1.15)	1.12	(0.74–1.69)
**Caste of household**				
Scheduled Tribes	1.00		1.00	
Scheduled Castes	1.19	(0.95–1.48)	0.50*	(0.23–1.06)
Other Backward Classes	1.19	(0.96–1.47)	0.44**	(0.22–0.90)
Others	1.08	(0.86–1.34)	0.40**	(0.19–0.83)
**Religion of household**				
Hindu	1.00		1.00	
Muslim	0.97	(0.82–1.13)	1.31	(0.81–2.11)
Others	1.16*	(0.97–1.38)	0.82	(0.51–1.30)
**Participates in social group**				
Yes	1.00		1.00	
No	1.03	(0.93–1.13)	1.26	(0.93–1.70)
**Place of residence**				
Urban	1.00		1.00	
Rural	1.05	(0.93–1.19)	0.81	(0.58–1.15)
**Country regions**				
Northern	1.00		1.00	
North Eastern	0.18***	(0.11–0.28)	3.29	(0.55–19.9)
Central	2.72***	(2.31–3.19)	0.51**	(0.29–0.91)
Eastern	0.75***	(0.65–0.87)	2.70***	(1.59–4.56)
Western	0.70***	(0.60–0.81)	1.83***	(1.18–2.82)
Southern	1.40***	(1.23–1.59)	0.79	(0.53–1.17)
**Number of older adults**	**9,533**		**994**	

Note – (a) Categories with odds ratio 1.00 are the reference category (b) * denotes *p*-value < 0.1, ** denotes *p*-value < 0.05, *** denotes *p*-value < 0.01 (c) OR: Odds ratio (d) 95 % Confidence interval (CI) is given in brackets

## Discussion

The present study uses the panel dataset of IHDS (2004-05 and 2011-12), which aims to capture the dynamics of older adults acquiring or getting recovered from disability. It was found that about 32 % of older adults acquired disability from 2004 to 05 to 2011-12 and about 4 % of older adults got recovered during the same period. Moreover, in 2011-12 nearly 37 % of older adults were disabled in comparison to 9 % in 2004-05. It was argued by WHO that the prevalence of disability among older adults is increasing drastically due to changing demographic trends, that is, an increase in the share of older adults and due to an increase in chronic health condition among older adults [48].

Older women had a higher likelihood of acquiring disability than older men. The results were consistent with the previous literature which argued that gender differences do exist as women develop disability more often than men do as their survival rates are higher than men [49]. Oldest-old had higher odds to acquire disability along with lower odds for recovering from disability than younger older adults. The results were parallel with the previous finding that the occurrence of disability increases with the ageing population [50]. Previous studies found an association of chronic morbidities with the prevalence of disability [51, 52], and a few studies concluded that chronic diseases contribute significantly to the procurement of disability among older adults [51, 53]. Consistently, the present study found that older adults with any chronic disease had a higher likelihood to acquire a disability.

Older adults with non-working status had higher odds to acquire a disability as compared to those with working status. The plausible reason is that older adults who were in non-working category were most likely to acquire the sedentary life style in later stages of life [54]. A sedentary life style lead to the acquisition of pathology (acute or chronic diseases, injury) and results in functional limitations which finally leads to disability [54]. Disability among older adults hinders the working status and hence poses an economic burden among older adults [55]. Marital status had a protective effect towards acquiring disability in older ages. The results are consistent with the previous findings [56]. Older adults co-residing may be dependent on others for household chores; hence being married was also associated with a higher chance of recovery. The older adults living in the single-generation household had a higher likelihood to acquire a disability. Similar findings were visible in the previous studies that older adults living in single generation households had a higher likelihood to suffer from the disability [57]. In a single-generation household, the older adults are not dependent on others to be taken care of financially nor in any household chores [57]. Yet, we cannot fully dismiss the probability that disability determines living arrangements rather than living arrangements impacting disability in the present research.

Further, present analysis found that the older adults who belonged to the richest household wealth quintile had a higher likelihood to recover from a disability. Earlier studies concluded that older adults from lower socio-economic classes had a higher risk for activities of daily living, instrumental activities of daily living, and functional limitations [39], indicating a higher prevalence rates and lower recovery rate due to health resource constraints. Moreover, a longitudinal analysis suggested that self-perceived income adequacy predicts the median age for onset of disability and lower levels of perceived income result in poor health outcomes among older adults [58]. Additionally, it was earlier argued that treatment-seeking was higher among older adults from the richest wealth quintile and higher castes in comparison to their counterparts with low socioeconomic status [40], possibly leading to lower recovery rates among low socioeconomic groups. On the other hand, low caste groups were found to be more likely to recover from disability and the odds were comparatively lower but insignificant in the case of acquiring disability over the period. This can partially be explained by the absence of a large portion base-line sample in the second round who could not survive and the higher recovery rate among those who survived both the rounds.

Older adults from the central and southern regions were more likely to acquire a disability in comparison to those from the northern region of India. The relationship is quite spurious in the case of southern India as most of the states from southern India tops in the health index score in a report by NITI Ayog (National Institution for Transforming India) [59]. However, the condition of health care infrastructure is not improving in the states like Madhya Pradesh, Uttar Pradesh, and Chhattisgarh which fall into the central Indian region had low rank in health index score [59]. The association also needs further investigation as it was found in previous studies that treatment-seeking was higher in southern states of India [40]. However, the probable reason for the higher occurrence of disability among older adults in southern India may be due to a higher proportion of older adults in the respective region [60].

In comparison to other literature from India, the present study backs different strengths. Firstly, we used the nationally representative database which contains prominent information on disability. Secondly, the panel nature of the dataset allows us to track the changes in disability status among the same individual and provide evidence of its association with different characteristics of the elderly. The results of this study emphasize from a public health perspective, the need of the hour to focus on the growing prevalence of disability across the Indian elderly. This study also had certain limitations. Although the panel sample is largely similar to the baseline sample by the individual and household socio-economic characteristics, the findings of our study are affected by attrition bias. Therefore, the results need to be interpreted carefully. Further, the disease taken into consideration were self-reported and may include reporting bias. Moreover, due to limited information on disability in the data, the study had restricted its analysis to certain physical limitations only. Additionally, the findings from our study do not claim causal effect. However, with the help of relevant data future studies need to examine whether change in determinants like chronic diseases, marital status, working status, etc. led to the transition in disability status among the older adults.

## Conclusions

The present study focused on acquiring and recovery from disability among older adults in India from 2004 to 05 to 2011-12. The study emphasized the importance of considering chronic physical diseases and several socio-demographic factors that may negatively affect the trajectory of disability in older ages. Older adults who had any chronic disease, were in non-working category, from single generation household and southern region should be in focus as they are at higher risk for acquiring the disability. Older adults from poorest household quintile should be prioritize and subsidized medical health facility should be provided so that they can go for treatment if acquired disability due to certain underlying causes. Even central region of India needs special attention for better medical facilities to older adults. Further, policymakers should develop joint programs focused on health promotion, and the development of disability prevention in the older population.

## Data Availability

The IHDS datasets used in our study can be downloaded from the Inter-University Consortium for Political and Social Research (ICPSR) data repository at http://www.icpsr.umich.edu.

## Abbreviations

OR: Odds Ratio
CI: Confidence Interval
IHDS: India Human Development Survey
OBC: Other Backward Classes
SC: Scheduled Caste
ST: Scheduled Tribe

## Declarations

### Author’s Information

Ronak Paul and Rashmi completed their M.Phil. in Biostatistics and Demography and currently pursuing their Ph.D. in Biostatistics and Demography from International Institute for Population Sciences, Mumbai.

Shobhit Srivastava and Muhammad T completed their M.Phil. in Population Studies and currently pursuing their Ph.D. in Population Studies from International Institute for Population Sciences, Mumbai.
